# Effect of a Single Nutritional Intervention Previous to a Critical Period of Fat Gain in University Students with Overweight and Obesity: A Randomized Controlled Trial

**DOI:** 10.3390/ijerph17145149

**Published:** 2020-07-16

**Authors:** Sam Hernández-Jaña, Tamara Huber-Pérez, Ximena Palma-Leal, Paola Guerrero-Ibacache, Valentina Campos-Nuñez, Juan Pablo Zavala-Crichton, Carlos Jorquera-Aguilera, Kabir P. Sadarangani, Fernando Rodríguez-Rodríguez, Carlos Cristi-Montero

**Affiliations:** 1 IRyS Research Group, School of Physical Education, Pontificia Universidad Católica de Valparaíso, 2374631 Valparaíso, Chile; sam.hernandez.jana@gmail.com (S.H.-J.); t.huberperez@gmail.com (T.H.-P.); ximena.palmaleal@gmail.com (X.P.-L.); pguerreroibacache@gmail.com (P.G.-I.); camposnunezvalentina@gmail.com (V.C.-N.); fernando.rodriguez@pucv.cl (F.R.-R.); 2 Faculty of Education and Social Sciences, Universidad Andres Bello, 2531015 Viña del Mar, Chile; jzavala@unab.cl; 3 School of Nutrition and Dietetics, Faculty of Science, Universidad Mayor, 8580000 Santiago, Chile; carlos.jorquera@mayor.cl; 4 Department of Kinesiology, Universidad Autónoma de Chile, 7500912 Santiago, Chile; kabir.sadarangani@gmail.com; 5 School of Kinesiology, Faculty of Health and Dentistry, Universidad Diego Portales, 8370057 Santiago, Chile

**Keywords:** weight gain, obesity prevention, critical periods, eating disorders, intervention

## Abstract

Background: the present study aimed to investigate the effects of a single nutritional preventive session previous to a critical period linked to fat gain in university students with overweightness and obesity, emulating a nutritional session of a public health system. Methods: In this single-blind randomized controlled trial, 23 students met all the criteria to be included (20.91 ± 2.52-year-old; 52.2% women) who were divided into two groups: intervention group (IG) and control group (CG). Fat mass (FM) by dual-energy X-ray absorptiometry (DXA), physical activity by accelerometry, feeding evaluation through three questionnaires, and a set of healthy lifestyle recommendations were evaluated before and after the national holidays (NH). Results: Our findings showed that FM increased significantly in the CG, but not in the IG (CG = 428.1 g; IG = 321.9 g; Δ = 106.2 g; *p* = 0.654 [95% CI = −379.57, 591.92]). However, no differences were found during the NH between them (Hedges’ g effect size = 0.19; *p* = 0.654). In addition, no statistical differences were observed between groups in feeding evaluations, the set of recommendations performed, and physical activity. Conclusion: a single preventive session before a critical period, using a similar counselling approach as used in the public health system, might not be enough to promote changes in eating and physical activity patterns and preventing fat gain in overweight/obese university students. Long-term interventions are a must.

## 1. Introduction

Overweightness and obesity are defined as an abnormal or excessive accumulation of fat that affects health severely [1]. Globally, overweightness and obesity have increased drastically in the last 20 years [2], affecting, in 2016, 39% of the adult population worldwide [1]. This pandemic contributes strongly to the development of different types of non-communicable diseases, such as type 2 diabetes, hypertension, some types of cancers, cardiovascular diseases, mental health, and eating disorders [3,4,5], increasing the global burden of disease significantly [6].

There are several causes associated with overweightness and obesity, but the main reason is the disproportion between calories consumed and calories expended [1]. Factors such as the expenditure of energy, level of physical activity, time on sedentary activities, and quantity and quality of food (input of energy) are essential for understanding and decreasing the risk of obesity [3]. A meta-analysis showed a differenced effect on body weight and visceral adipose tissue with exercise training or a hypocaloric diet [7]. Nonetheless, today it is undeniable that both strategies must be included in an intervention against obesity in any period across the lifespan [8].

In this sense, a relevant period of life in which physical activity levels and dietary behavior change abruptly is during the university stage [9,10]. As a result, students increase their weight and fat mass (FM) significantly [11]. Therefore, it is vital to implement multidisciplinary and preventive actions on this population to face this issue [11,12,13], also considering that most of their daily time is spent at universities.

In another context, it has been established that during the year, there are many critical periods in which the population is more likely to gain weight, such as winter or summer holidays, Thanksgiving, new year, and national holidays (NH). These periods are characterized by changes in behavior, specifically in eating and physical activity [10,14], which explains the fluctuation observed in weight and FM. This accentuated variation on body composition, attained in a couple of days or weeks, induces a harmful physiological environment (i.e., a high risk of low-grade systemic inflammation, affecting insulin signaling, etc.), an effect that could even last for several years [15]. Furthermore, the weight and FM gained during a critical period do not seem to be offset before the next critical period, forming a “ladder effect” [16]. Then, prior interventions to critical periods are essentials in the general population, but seem to be especially necessary for university students.

In our national context, Chile is ranked as one of the most obese countries worldwide [17]. Currently, 74.2% of the adult population present as overweight or obese [18], exceeding the world average by more than 30%. A recognized critical period linked to gain weight in Chile is the NH. During this holiday, that lasts between seven and nine days, students increase the consumption of high-calorie foods, while physical activity levels decrease [9,16]. In this line, two previous studies in schoolchildren have shown an increase in body weight (0.250 to 0.692 kg) and percentage of fat (about 2.2%) [16,19]. To counteract this scenario, a study carried out a nutritional intervention in Chilean schoolchildren previous to the NH, finding no significant increases in body weight after two short preventive workshops in comparison with a control group [19]. Thus, brief talks on eating behaviors and healthy lifestyle appear to help with weight management in this age group during a critical period; however, could this kind of intervention be useful in university students with overweight and obesity? To date, these types of interventions have not been contrasted in the university population.

In addition to the before-mentioned evidence linked to behavioral interventions in this population is scarce and necessary [11,14], and few studies have tested the effectiveness of a short-term strategy, as used in the public health system. Therefore, the objective of this study was to examine the effect on FM of a single nutritional preventive session before the NH in Chilean university students with overweightness/obesity.

## 2. Materials and Methods

### 2.1. Study Design

**Figure 1:**
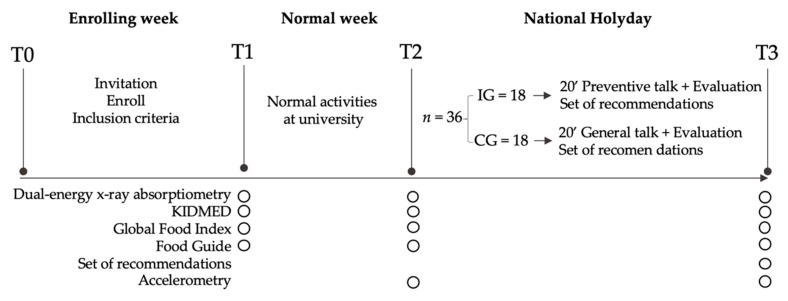
Study design; T: evaluation time; IG: intervention group; CG: control group; KIDMED: Mediterranean Diet Quality Index.

A single-blind randomized controlled trial was carried out, over a duration of three consecutive weeks during September 2017. This study was conducted according to the Consort Statement guidelines and their checklist can be found as Supplementary Material 1 [20]. The deontological norms of the Declaration of Helsinki for studies in human beings were respected. The project was approved by the Ethics Committee of the Pontificia Universidad Católica de Valparaíso (BIOEPUCV-H145-2017). Figure 1 shows the study design.

### 2.2. Participants

The sample size was calculated based on a previous study from our research team [19], accepting an alpha risk of 0.05, a beta risk of 0.2 in a bilateral contrast, type of test = two-sided, and a dropout rate of 0.20 (20%). Therefore, at least 18 subjects were necessary for each group. All participants belonged to the Andrés Bello University (UNAB), Viña del Mar. During the enrolling week, a stand was placed in the university, where students were invited to participate (by J.P.Z.-C). A total of 148 students were enrolled voluntarily, but only 48 met all inclusion criteria (body mass index—BMI ≥ 25 kg/m^2^, not having metal plates, not studying nutrition and dietetics or physical education, and not being pregnant) (T0, Figure 1).

**Figure 2:**
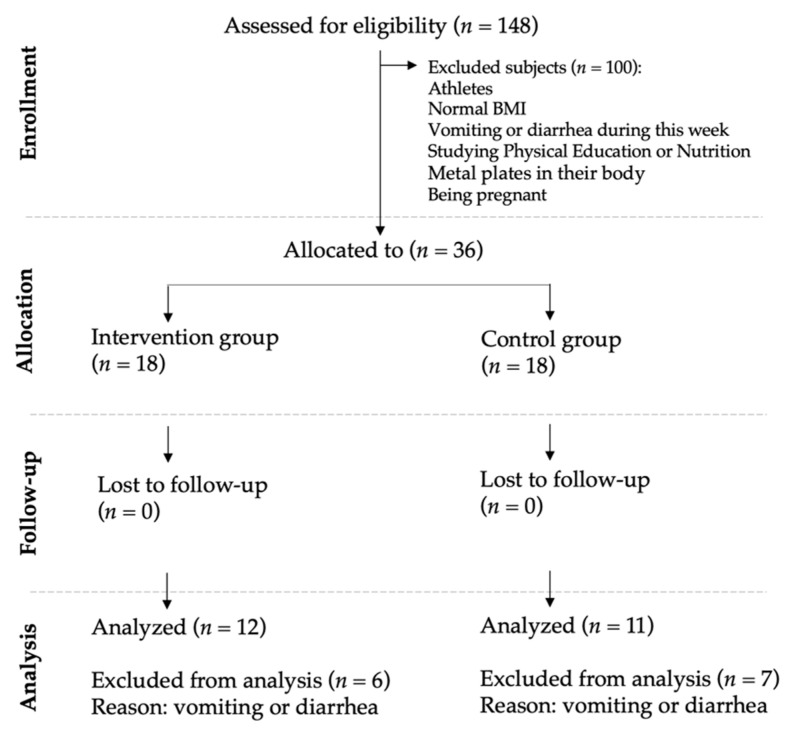
Consort diagram of the participant flow.

Before the randomization, allocation concealment was implemented to avoid randomization bias. The assignation to the intervention or control group was not performed until just before starting the second session (T2, Figure 1). After the allocation concealment, a researcher (C.C.-M) who did not participate in the enrolling period, performed the randomization. A total of 36 subjects (50% men and 50% women) were randomized and split into two groups of 18 participants (IG: intervention group and CG: control group) (https://www.randomizer.org). It is important to note that the group assignation was communicated to the evaluator only a couple of hours previously starting the intervention session (T2, Figure 1). Some participants presented gastrointestinal problems (vomit or diarrheas) during the three weeks of the experiment; thus, 13 participants had to be removed. Figure 2 shows the flow diagram of the progress through the study phases. Due to this high rate of exclusion, we performed an intention-to-treat analysis (Supplementary material S2).

### 2.3. Procedures and Instrumentation

All measurements (general participants’ characteristics, body composition, feeding questionnaires, accelerometry, and set of recommendations) were evaluated in the Physical Education School Laboratory for two weeks. Participants were randomized and blinded to the nutritional intervention session.

### 2.4. Body Composition Measurements: Primary Outcome

Body weight was measured with a digital scale (TANITA, Model HD-313, Tokyo, Japan) and height with a stadiometer (SECA, model 213, GmbH, Germany) with precisions of 100 g and 1 mm, respectively. BMI was calculated as the weight in kilograms divided by the square of height in meters (BMI = kg/m^2^). This measure was only used as an inclusion criterion (T0, Figure 1).

FM was evaluated through a dual-energy x-ray absorptiometer (iDXA Scan, General Electric, Madison, WI, USA). Each participant was asked to lie down on the DXA stretcher and the whole procedure lasted 7 min and 16 s (radiation dose of 3.0 µGy, voltage 100.0 kV, and current 0.188 mA). This measure was evaluated at three-time points (T1, T2, and T3, Figure 1) and at the same time every day, avoiding weight fluctuation due to meals.

### 2.5. Feeding Questionnaires and Food Guide Recommendations: Secondary Outcome

Three questionnaires were used to evaluate changes in dietary patterns.

Global food index (GFI) measures the compliance of university students to the food recommendations [21]. This frequency of daily and weekly food consumption survey evaluated 12 items: 5 of them about healthy foods, 4 about unhealthy food, and the remaining 3 about their main meals. Each variable scored from 1 to 10, where 1 is less healthy and 10 corresponds to compliance with the recommendation. The total score was divided into three categories: (1) “unhealthy diet” (<60), (2) “needs changes” (60–89), and (3) “healthy diet” (90–120) [21].

The KIDMED (Mediterranean Diet Quality Index) is a questionnaire that establishes the adherence to the Mediterranean diet. It consists of 16 items separated into two sections. The first section has 4 questions related to negative adherence to the Mediterranean diet (consumption of fast food, baked goods, sweets, and skipping breakfast), scoring −1 point when present; meanwhile, the second section has 12 questions based on positive relation, scoring +1 point for the following consumptions: oil, fish, fruits, vegetables, cereals, nuts, pulses, and dairy products. The total score generates three categories, “poor adherence” (0–3), “average adherence” (4–7), and “good adherence” to the Mediterranean diet (8–12) [22].

Lastly, the food guide recommendation from the Ministry of Health of Chile, created in 2013, was evaluated. This guideline is a set of 11 educational messages that adapt scientific knowledge about diet and physical activity to information which needs to be obtained in the general population, considering their health situation and sociocultural factors. It aims to evaluate population compliance [23].

### 2.6. Physical Activity Level: Seconday Outcome

Physical activity levels were measured with accelerometers (GT3X+, ActiGraph Inc., Pensacola, USA). Each participant wore an accelerometer on their waist for 14 days (T1-T2 and T2-T3, Figure 1). This device was only removed when taking a shower, sleeping, or when practicing water activities such as swimming. Each accelerometer was set at 90 Hz, 1 min duration by epoch, and to establish the physical activity intensity, the Freedson’s algorithm was used [24]. Then, a range cut point of 0–99, 100–2019, ≥2020 were used to determine a sedentary activity, light-intensity physical activity (LIPA), and moderate-to-vigorous physical activity (MVPA), respectively. A valid register was established when the device was used a) a minimum of 10 h per day and b) at least three days of a weekday and one day of the weekend during a 7-day period [25].

### 2.7. Intervention

An intervention was performed, emulating a traditional nutritional session from a primary healthcare center (public health system). Traditionally, this session lasts around 20 min and includes a body composition measurement, nutritional assessment, and a brief educational talk about healthy eating (T2, Figure 1). As in other countries in the world, specific recommendations are made during the last nutritional session, when a critical period linked to weight gain is approaching. In our study, the only difference with the CG was that the IG group received a series of healthy recommendations specially focused on the NH; the CG was asked to continue their normal activities.

In detail, nutrition specialists carried out a single educational talk, which included two parts. The first part involved information about obesity health risks, the benefits of a healthy lifestyle (nutrition and physical activity pieces of advice), distribution of meals, and the detrimental effect of a critical period such as the NH. In the second part, six recommendations were delivered: (1) to decrease food intake during each meal, (2) to reduce alcohol and sugary beverage consumption, (3) to increase physical activity levels, by dancing or playing national games, (4) to do not add mayonnaise, ketchup or salt to foods, (5) to consume only one typical food per day, and (6) to increase the ingest of vegetables. These recommendations arose from a committee of specialists conformed previously at the beginning of this study (T3, Figure 1).

### 2.8. Statistical Analysis

The normality of the data distribution was tested using Shapiro–Wilk test. Continuous variables are presented as mean and standard deviation (SD). A *t*-test was performed to compare differences between groups before beginning the intervention (Table 1). ANOVA was used to determine the interaction between intervention × sex and intervention × age. Repeated measures ANOVA were used to compare the different evaluation times (T1, T2, and T3) per intervention group (CG and IG). Bonferroni post-hoc was used to establish differences among groups per each time.

A *t*-test was used for independent samples to compare differences between deltas (post- and pre-values of FM, physical activity, and feeding questionnaires) by groups, and the Wilcoxon test was used for paired samples when normality was violated. In addition, the Chi-square and Fisher’s exact tests were used to compare variation on nutritional questionnaires and set of dietary recommendations. Hedges’ g, which includes a correction for small sample bias, was used to estimate the effect size, complementing the *p*-value from *t*-tests. Hedges’ g was interpreted as no effect (<0.2), small (0.2 to <0.5), medium (0.5 to <0.8), and large (≥0.8) [26]. The significance level was set at *p* < 0.05. Finally, a sensitivity analysis was performed for intention-to-treat (supplemental material 2).

## 3. Results

The final sample was composed of 23 participants after applying the unique exclusion criteria (CG = 11 and IG = 12; 12 women and 11 men). Thus, 13 participants were excluded (0.36 dropout rate) for presenting vomit or diarrhea. No interaction between sex (*p* = 0.245) and age (*p* = 0.219) was found; thus, the analysis was not adjusted for these variables. Table 1 shows the baseline characteristics of each group. Overall, only one statistical difference was found in the food guide recommendation (*p* = 0.049 [95% CI = −3.49, −0.01]); however, no differences were found in any variable when sensitivity analysis was performed (Supplemental Material 2).

### 3.1. Fat Mass

**Figure 3:**
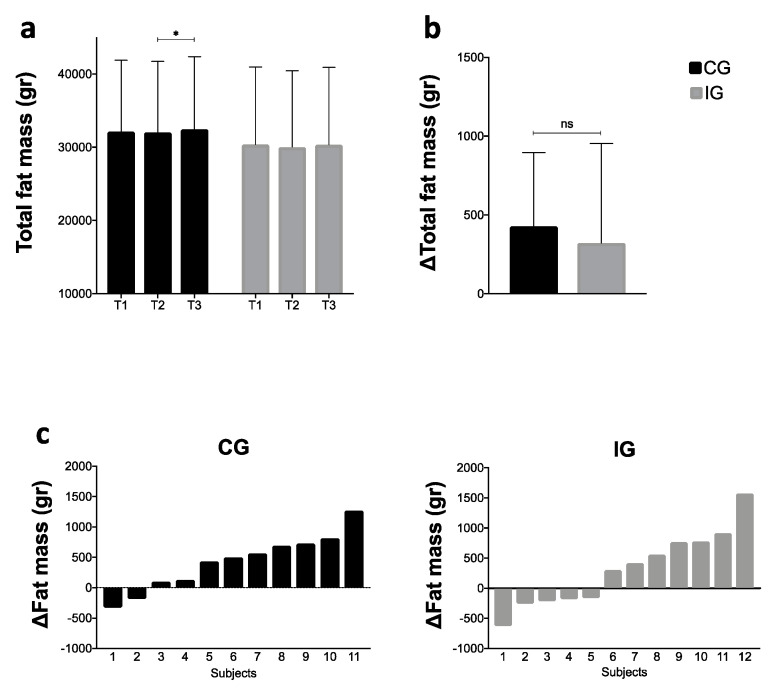
(**a**) Intra-group values of total fat mass during T1, T2, and T3; (**b**) comparison of the total FM variation between groups during the NH (T3−T2); (**c**) individual fat mass variation during the NH by groups. CG: control group; IG: intervention group; NH: national holidays; * Statistical significance (*p* < 0.05); NS: no statistical significance.

Figure 3 shows total FM (T1, T2, and T3), variation of FM among groups during the NH (critical period), and individual variation by groups during the NH. Figure 3a shows that CG had a significant increase in the total FM during the NH (1.30%; Δ = 428.1 g; Hedges’ g = 0.04; *p* = 0.038 [95% CI = 23.58, 832.60]), while the IG group did not (1.08%; Δ = 321.9 g; Hedges’ g = 0.03; *p* = 0.316 [95% CI = −192.37, 836.21]). The sensitivity analysis also showed a significant difference between T1 and T3, but this finding must be taken with caution due these participants being excluded by gastrointestinal problems, an important exclusion criterion in these kinds of studies.

Figure 3b showed no differences between the groups during the NH (Hedges’ g = 0.19; *p* = 0.654 [95% CI = −379.57, 591.92]). Figure 3c shows the individual variation of FM by groups during the NH. CG’s FM variation fluctuated between −0.79% and 2.78% (two participants decreased their FM and nine increased their FM), while IG’s FM variation fluctuated between −1.83% and 5.77%, (five participants decreased their FM while seven increased their FM).

### 3.2. Nutritional Questionnaires

**Figure 4:**
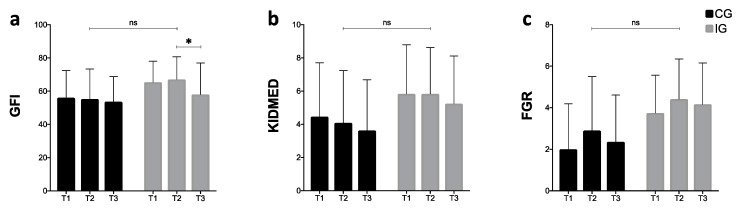
Intra and inter-group values between CG and IG at T1, T2, and T3. (**a**) GFI: global food index; (**b**) KIDMED: Mediterranean diet quality index; (**c**) FGR: food guide recommendations; CG: control group; IG: intervention group; NH: national holidays; * Statistical significance (*p* < 0.05); NS: no significance.

Figure 4 shows scores obtained from three different questionnaires in each evaluation time. The global food index (Figure 4a) displays a continuous decrease in the CG, while a significant reduction in the score after the NH is observed in the IG (*p* = 0.022 [95% CI = −16.91, −1.26]). Regarding the KIDMED results (Figure 4b), both groups presented a decreasing trend during the three weeks. Finally, the food guide recommendations (Figure 4c) show the same behavior between groups, increasing the score in T2, but decreasing in T3. Overall, there were no statistical differences between the groups in any questionnaire evaluated.

Table 2 shows the results of the adherence to the set of recommendations between the groups during the NH. Only one statistically significant difference was found in compliance with the recommendations regarding increasing physical activity levels (*p* = 0.003).

### 3.3. Accelerometry

**Figure 5:**
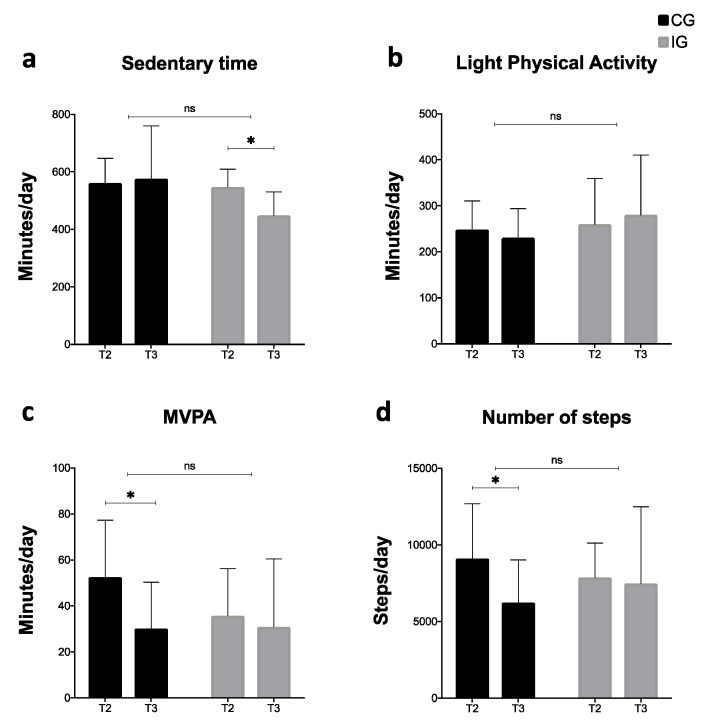
Intra and inter-group physical activity comparisons. MVPA: Moderate to vigorous physical activity; CG: control group; IG: intervention group. NH: national holidays; * Statistical significance (*p* < 0.05); NS: no significance.

Figure 5 shows the results related to time spent by sedentary activities; LIPA, MVPA, and the numbers of steps were obtained by accelerometry. Only 17 of the 23 participants had valid data for the analysis (CG = 10 and IG = 7). Sedentary time was reduced in the IG significantly during the NH (*p* = 0.016 [95% CI = 47.10, 145.00]), but not in the CG. However, LIPA did not display significant variations in both groups. MVPA decreased in both groups, but only the CG reached a significant variation (*p* = 0.003 [95% CI = −35.13, −9.57]). Finally, the number of steps decreased in both groups; however, statistically significance was only present in the CG (*p* = 0.011 [95% CI = −4929.88, −828.33]). Despite some intra-group differences, overall, there were no physical activity differences between groups.

## 4. Discussion

The main result of this study was that there were not a significative effect on body composition, nutritional state, and physical activity using a single preventive intervention previous to a known critical period in Chile in a clinical population of university students with overweightness/obesity. Overall, this study helps to increase the little existing evidence on lifestyle interventions in a group that represents around 56.7% of the total students enrolling in higher education in Chile [27]. In addition, this study helps to determine whether the way of facing a critical period linked to weight gain by the public health system is the most adequate.

The primary outcome of this current study showed that there was no significant effect on preventing FM increase using a single nutritional session before a critical period. To our knowledge, to date, there is no evidence of this sort of intervention in overweight/obese university students previous to an avowed critical period, for instance, Thanksgiving, new year, or the NH. In this sense, the unique and similar study to the present intervention is one published by our research team in schoolchildren, which, in turn, supports the current proposal. This study found a significant effect of preventing body weight gain using a brief preventive intervention before the NH [19]. Unlike university students, children and adolescents may be more receptive to this sort of intervention, due to their behavior being influenced more strongly by the family environment [28] and their eating behavior is not yet as ingrained. Thus, a single nutritional preventive intervention in the university population may not be enough to guarantee changes in dietary and physical activity habits, preventing fat gain, unlike in schoolchildren.

A study that confirms the aforementioned found that despite university student knowing the importance of warning labels on food consumption, they showed strong pre-contemplative stages and resistance before changing their consumption habits [29]. These results clearly show the high degree of rooted eating habits in this population, but in particular, in people with overweightness or obesity [30]. In this line, a panel of experts in the management of overweightness and obesity in adults mentioned that at least 14 sessions are necessary within the first 6 months to promote lifestyle changes and obtain the best results [31].

Regarding the variation of the body composition in the present study, the total FM tended to increase after the NH in both groups (1.08 vs. 1.30%), but only the CG group result was statically significant. Nevertheless, there was no statistical difference between them. Although this percentual variation does not seem important at first glance, a systematic review and meta-analysis showed that during college (5 years), students increased their weight by 1.55 kg and FM in 1.17%, independently of their sex and baseline BMI [11]. In this way, critical periods are crucial for the drastic body composition modification during the year [14], increasing the risk of obesity.

Our study showed that nine days were enough to increase FM over 1%. Similar to these findings, a study found that during Thanksgiving, university students with a normal BMI increased by 3.4% their FM, measured by DXA. In contrast, overweight and obese students increased their FM by 5.0% [9]. Moreover, another study in the United States showed that the winter holiday season contributes to over half of annual body weight gain, primarily from increased food intake [32]. Therefore, as it has been mentioned in their conclusions [11], it is highly necessary to design prevention strategies for the college population, which could mediate positively in reducing adults overweight and obesity rates.

Concerning the nutritional evaluation through questionnaires (secondary outcome), results obtained in both groups showed a decreasing trend towards the consumption of a healthy diet after the NH, in both groups. The GFI has already been evaluated in Chilean university students, showing severe deficiencies in dietary quality, depicting only 9.3% of adherence to a healthy diet [21]. This outcome, by Ratner et al., corroborates our findings regarding the poor eating habits of this population. In relation to KIDMED, the level of adherence to a Mediterranean diet decreased during the NH. Studies declared that those university students with low adherence to the Mediterranean diet have a higher risk of being overweight [33,34]. They have been linked to poor consumption of cereals, legumes, fish, fruits, and vegetables, displaying a significant imbalance on their caloric food intake. All of these results support the protective role of a balanced diet, such as the Mediterranean diet, on weight status [33,34]. Finally, the Chilean food guide recommendations indicated a low consumption of dairy products and high consumption of sugary processed drinks and juices, contradicting the recommendations by international and national organisms [35]. The findings of this study strengthen the importance of developing multidisciplinary activities that promote healthy eating at universities, preventing obesity and their associated diseases.

Regarding the physical activity evaluation (secondary outcome), we observed a reduction in MVPA levels and the daily number of steps in the CG, and a reduction in sedentary time in the IG. Although there were intra-group changes, no statistical differences were found between the control and intervention groups, indicating that the intervention did not yield the expected impact. Despite the scarce evidence on lifestyle interventions in this population [14], a study investigated the effect of exercise changes on body composition during 5 years at university. They found a pronounced decrease in physical activity levels (around 33%) and particularly a reduction in exercise intensity in students without exercise intervention. These results were associated with increased body mass and FM [36]. Although the long term effect of the body composition leads to an increased risk of diverse diseases, a short-term physical activity reduction, as during a critical period, can generate modifications in the body composition and metabolic health [15,37], alterations which could even last for several years [15].

According to other studies, lack of time is a primary reason for physical inactivity in university students [34,38]. However, during holidays (e.g., Thanksgiving, new year, or the NH), students have more leisure time to spend on these types of activities, but physical activity levels do not increase. This scenario demonstrates, to some extent, the lack of healthy lifestyle habits in this population, and also reflects the protector effect of educational establishments, such as universities, regulating obesogenic behaviors in theirs students: this, in the literature, is known as the “Structured Days Hypothesis” [39].

Overall, an unfavorable panorama is observed during the NH, which involves a reduction in physical activity levels, adverse dietary habits, and an increase in FM. All these factors yield an excessive volume of adipose tissue, modifying the function and number of adipose cells. This response of the organism causes a local or low-grade systemic inflammation [40], which, in turn, is correlated with the development of cardio-metabolic diseases [41] and cancers [40]. Therefore, it is recommendable that critical periods should be taken into account in the public health system as a potential hazard to populations health, because the sum of short critical periods during a year could affect people’s health in the future. Thus, two points are important to highlight: The first is that intervention in overweight/obese university students focusing on acquiring positive eating habits and lifestyles should be prioritized [29,34,42]. The second is that the current way to face a critical period by the Chilean public health system neither guarantees nor promotes modification on eating and physical activity behavior.

### Strengths and Limitations

This study presents some limitations, for instance, the reduced number of final participants due to gastrointestinal problems and the difficulty of wearing an accelerometer for two weeks in a row. Thus, many of them were excluded for not meeting the analysis criteria, which, in turn, can be a strength, considering the higher reliability of the data obtained. In addition, the final sample size did not permit us to include some covariates in our analysis. Thus, our findings must not be extrapolated to the entire university population, and it is recommendable that to be considered an exploratory study.

One remarkable strength of this study is that FM was evaluated three times through a dual-energy x-ray absorptiometer (gold standard), which increases the accuracy of results in our assessment. In addition, to the best of the authors’ knowledge, this is the first study which evaluates the effect of a single and short nutritional intervention previous to a critical period in university students with overweightness/obesity. The last reinforces some recommendations emanating from systematic reviews and meta-analyses in this area, restating the necessity to test new interventions in this population and from other countries, due to the fact that most of the studies come from the United States [14]. Finally, this intervention emulated a primary care nutritional appointment as a more ecological fashion, to transfer our findings.

## 5. Conclusions

In conclusion, a single and short-term nutritional intervention before a critical period might not have a significant effect on preventing FM gain in university students with overweightness/obesity. These findings partially show, on the one hand, strong adherence to lifestyles that are already rooted in students. On the other hand, long-term interventions to revert the impact on body composition during a critical period seem to be essential because the current public strategy neither guarantees nor promotes modification on eating and physical activity behavior. These findings must be considered with caution due to the high rate of participant exclusion and therefore, their reduced final sample size.

## Figures and Tables

**Table 1:** Baseline characteristics of participants.

Variables	All (*n* = 23)	CG (*n* = 11)	IG (*n* = 12)	*p*-Value
Age (years)	20.91 ± 2.52	20.00 ± 1.41	21.75 ± 3.05	0.097
Weight (kg)	84.88 ± 14.88	85.04 ± 12.95	84.74 ± 17.03	0.963
Height (cm)	167.18 ± 10.18	165.15 ± 7.50	169.04 ± 12.18	0.372
BMI (kg/m^2^)	30.31 ± 4.26	31.14 ± 4.11	29.55 ± 4.42	0.383
Fat mass (kg)	31.20 ± 9.99	32.10 ± 9.78	30.37 ± 10.55	0.687
Global Food Index	60.98 ± 14.88	56.14 ± 16.23	65.42 ± 12.60	0.139
KIDMED	5.17 ± 3.10	4.46 ± 3.24	5.83 ± 2.95	0.297
FGR	2.91 ± 2.15	2.00 ± 2.19	3.75 ± 1.82	0.049 *
Accelerometry	All (*n* = 17)	CG (*n* = 10)	IG (*n* = 7)	
Sedentary time (min/day)	555.48 ± 75.32	560.83 ± 86.39	547.84 ± 61.79	0.739
LIPA (min/day)	253.15 ± 77.05	248.50 ± 62.28	259.80 ± 99.63	0.777
MVPA (min/day)	45.62 ± 23.97	52.52 ± 24.78	35.76 ± 20.46	0.162
Steps (steps/day)	8606 ± 3073	9118 ± 3568	7875 ± 2241	0.429

Mean ± SD. CG: control group; IG: intervention group; BMI: body mass index; KIDMED: adherence to the Mediterranean diet; FGR: food guide recommendation; LIPA: light-intensity physical activity; MVPA: moderate-to-vigorous physical activity; *p*-value compares IG and CG; * Statistical significance (*p* < 0.05).

**Table 2:** Adherence to recommendations after the NH.

Recommendations	CG		IG		*p*-Value
	No	Yes	No	Yes	
Decrease food intake.	4	7	4	8	1.000 ^†^
Decrease sugary drinks and liquor.	7	4	8	4	1.000 ^†^
Increase the level of physical activity.	10	1	3	9	0.003 ^†,^*
Do not add mayonnaise, ketchup, mustard, etc.	3	8	8	4	0.059 ^‡^
Eat one typical food per day.	4	7	2	10	0.371 ^†^
Increase salad intake.	2	9	7	5	0.089 ^†^

^†^ Fisher exact test; ^‡^ Chi-squared; CG: control group; IG: intervention group; * Statistical significance.

## Supplementary Materials

The following are available online at https://www.mdpi.com/1660-4601/17/14/5149/s1, S1: CONSORT 2010, Checklist. Table S1: Baseline characteristics of participants, Table S2: Adherence to recommendations after the NH., Figure S1: (a) Intra-group values of total fat mass during T1, T2 and T3; (b) comparison of the total FM variation between groups during the NH (T3-T2); (c) individual fat mass variation during the NH by groups., Figure S2: Intra and inter-group values between CG and IG at T1, T2 and T3, Figure S3: Intra and inter-group physical activity comparisons.
